# Neurophysiological Changes After Paired Brain and Spinal Cord Stimulation Coupled With Locomotor Training in Human Spinal Cord Injury

**DOI:** 10.3389/fneur.2021.627975

**Published:** 2021-05-10

**Authors:** Timothy S. Pulverenti, Morad Zaaya, Monika Grabowski, Ewelina Grabowski, Md. Anamul Islam, Jeffrey Li, Lynda M. Murray, Maria Knikou

**Affiliations:** ^1^Klab4Recovery Research Laboratory, Department of Physical Therapy, College of Staten Island, The City University of New York, New York, NY, United States; ^2^Ph.D. Program in Biology and Collaborative Neuroscience Program, Graduate Center of the City University of New York and College of Staten Island, New York, NY, United States

**Keywords:** H-reflex, locomotor training, neuromodulation, paired associative stimulation, rehabilitation, spinal cord injury, transspinal stimulation, transcranial magnetic stimulation

## Abstract

Neurophysiological changes that involve activity-dependent neuroplasticity mechanisms via repeated stimulation and locomotor training are not commonly employed in research even though combination of interventions is a common clinical practice. In this randomized clinical trial, we established neurophysiological changes when transcranial magnetic stimulation (TMS) of the motor cortex was paired with transcutaneous thoracolumbar spinal (transspinal) stimulation in human spinal cord injury (SCI) delivered during locomotor training. We hypothesized that TMS delivered before transspinal (TMS-transspinal) stimulation promotes functional reorganization of spinal networks during stepping. In this protocol, TMS-induced corticospinal volleys arrive at the spinal cord at a sufficient time to interact with transspinal stimulation induced depolarization of alpha motoneurons over multiple spinal segments. We further hypothesized that TMS delivered after transspinal (transspinal-TMS) stimulation induces less pronounced effects. In this protocol, transspinal stimulation is delivered at time that allows transspinal stimulation induced action potentials to arrive at the motor cortex and affect descending motor volleys at the site of their origin. Fourteen individuals with motor incomplete and complete SCI participated in at least 25 sessions. Both stimulation protocols were delivered during the stance phase of the less impaired leg. Each training session consisted of 240 paired stimuli delivered over 10-min blocks. In transspinal-TMS, the left soleus H-reflex increased during the stance-phase and the right soleus H-reflex decreased at mid-swing. In TMS-transspinal no significant changes were found. When soleus H-reflexes were grouped based on the TMS-targeted limb, transspinal-TMS and locomotor training promoted H-reflex depression at swing phase, while TMS-transspinal and locomotor training resulted in facilitation of the soleus H-reflex at stance phase of the step cycle. Furthermore, both transspinal-TMS and TMS-transspinal paired-associative stimulation (PAS) and locomotor training promoted a more physiological modulation of motor activity and thus depolarization of motoneurons during assisted stepping. Our findings support that targeted non-invasive stimulation of corticospinal and spinal neuronal pathways coupled with locomotor training produce neurophysiological changes beneficial to stepping in humans with varying deficits of sensorimotor function after SCI.

## Introduction

The pathological reorganization of spinal networks and disruption of supraspinal inputs after spinal cord injury (SCI) impairs modulation of muscle spindle reflexes which are partly responsible for the coordinated muscle activity during walking (1–6). For example, the electrical and mechanical evoked ankle Hoffmann (H) and stretch reflex are modulated in a pathological manner after SCI, with lack of stable motoneuronal depolarization during the stance phase and inhibition during the swing phase being the most common pathological behaviors (7, 8). Similarly, reduced intracortical inhibition and latency—amplitude of motor evoked potentials (MEP) support for impaired function of cortical and corticospinal neuronal activity after SCI (9, 10). Locomotor training partially restores the pathological behavior of ankle soleus H-reflex, corticospinal excitability, and motor cortex area activation upon active toe movement through reorganization of spinal interneuronal and corticospinal networks in human SCI (11–15).

Neurophysiological changes following combined interventions that involve activity-dependent neuroplasticity mechanisms via repeated stimulation and locomotor training are not commonly employed in research even though combination of interventions is a common clinical practice. Paired associative stimulation (PAS) of two different neural sites such as transcranial magnetic stimulation (TMS) of motor cortices (M1) or TMS of M1 paired with peripheral electrical nerve stimulation produces significant neuroplasticity (16, 17). This plasticity shares similar neuronal mechanisms to that of motor learning and exercise (18–20). In fact, when PAS is applied during repetitive motor tasks, the effects from PAS and learning from movement repetition are enhanced compared either one in isolation (21–24). Pairing TMS with transcutaneous spinal (transspinal) stimulation may have widespread neuroplasticity effects in human SCI. In principle, transspinal stimulation activates afferent and motor root fibers that transynaptically excite motoneurons and interneurons resulting in multisegmental transspinal evoked potentials (TEPs) susceptible to similar spinal inhibitory mechanisms as the soleus H-reflex (25). Further, we have recently shown that TEPs summate with the homonymous MEPs and soleus H-reflex in the surface electromyogram (EMG) (26, 27). Based on these findings we theorize that transspinal stimulation can activate dormant spinal networks and increase their sensitivity to residual supraspinal and sensory inputs enabling neuronal integration of signals after SCI in humans similar to that shown in anesthetized neurologically intact monkeys (28).

Repeated transspinal stimulation (one session) increases the MEP amplitude, decreases the afferent-mediated MEP facilitation and alters the subthreshold TMS-mediated flexor reflex facilitation in healthy subjects (29). When transspinal is paired with TMS, significant changes in intracortical, corticospinal, and spinal reflex excitability are evident (30). The effects were dependent on the relative timing between transspinal stimulation and TMS, with increased intracortical facilitation and corticospinal excitability when transspinal stimulation was delivered before TMS, while when TMS was delivered before transspinal stimulation corticospinal excitability was decreased (30, 31).

Taken altogether, we established the effects of locomotor training coupled with TMS and transspinal stimulation on the soleus H-reflex modulation pattern during assisted stepping and H-reflex excitability at rest in people with SCI. TMS and transspinal stimulation were delivered in a PAS paradigm during the stance phase of the less impaired leg at each step training session. TMS was delivered before (TMS-transspinal PAS) or after (transspinal-TMS PAS) transspinal stimulation. We hypothesized that TMS-transspinal PAS and locomotor training promotes a more physiological soleus H-reflex modulation pattern during stepping and H-reflex excitability at rest by strengthening spinal synapses largely due to the convergence of the two stimuli on spinal alpha motoneurons. We further hypothesized that transspinal-TMS PAS and locomotor training will produce less pronounced effects because transspinal stimulation before TMS affect spinal networks before descending motor volleys reach the spinal cord.

## Materials and Methods

### Participants

Volunteers were considered eligible if they met the following inclusion criteria: aged 18–75 years; diagnosis of first time SCI due to trauma, vascular, or orthopedic pathology; chronic (> 12 months) C2 – T11 SCI; presence of Achilles tendon reflexes; and hip and leg bone mineral density T score > 1.5. Exclusion criteria for the study included: presence of supraspinal lesions; neuropathies of the peripheral nervous system; presence of pressure sores; presence of medical implants (e.g., cochlear implants, pacemakers, baclofen pumps, etc.) and presence of implanted metals that are not MRI-safe; degenerative neurological disorders; and history of seizures. Eligible consented participants were asked to refrain from caffeine, alcohol and strenuous exercise for 12 h before the first test. Further, participants were asked to refrain from recreational drugs for 2 weeks before the first test. All experimental and training procedures were performed in compliance with the Declaration of Helsinki after a written informed consent was obtained from all participants before study enrollment. The experimental protocol was approved by the Institutional Review Board (IRB) of the City University of New York (IRB No: 2017-0261) and registered on ClinicalTrials.gov (NCT04624607).

### Study Design

**Figure 1:**
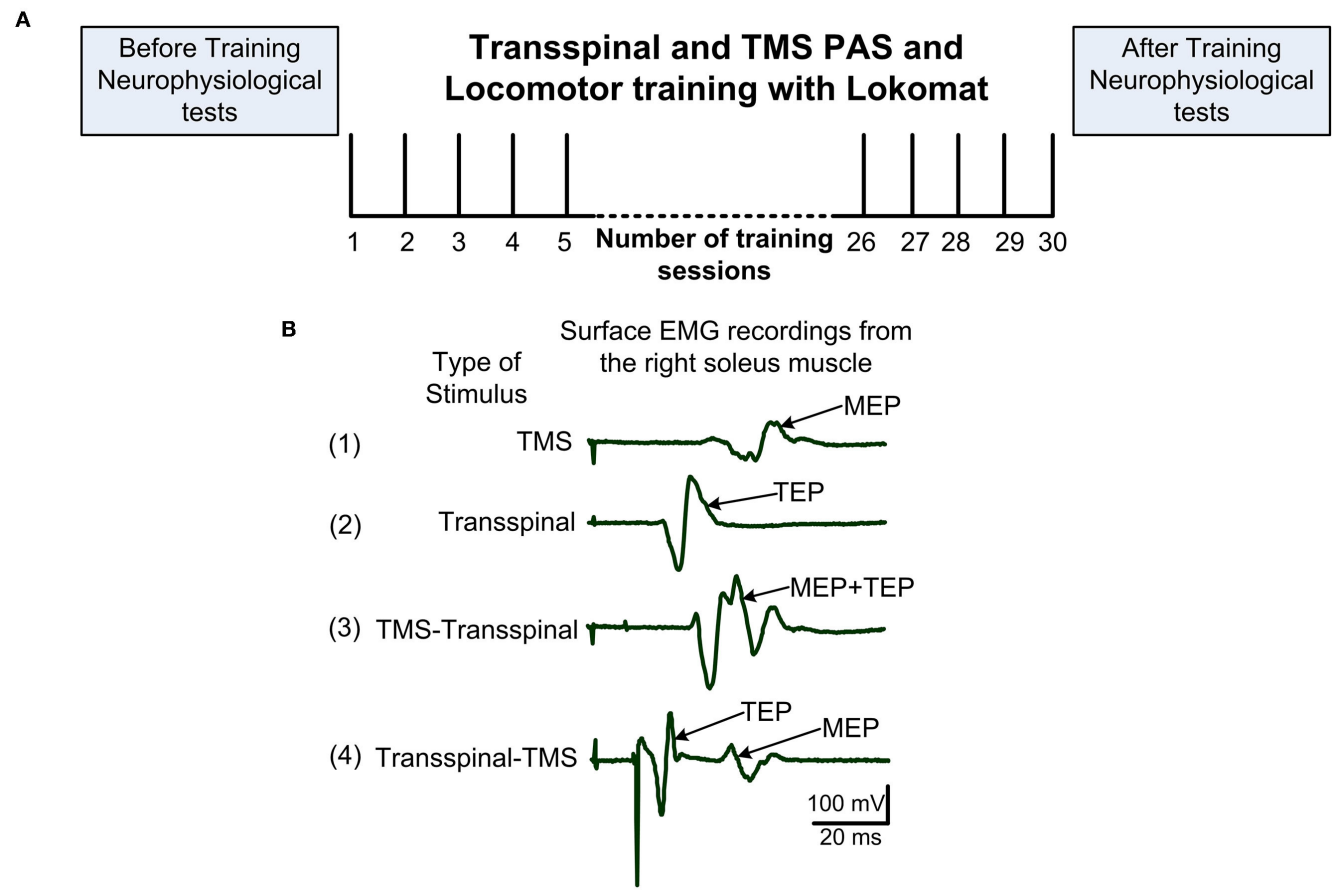
Experimental procedures. **(A)** After eligibility to the study was established, each subject was randomized to receive transspinal and transcranial magnetic stimulation (TMS) coupled with locomotor training via the Lokomat 6 Pro®. Before and 1-day after training neurophysiological tests were performed. Training sessions were delivered 5 days/week and 30 sessions were targeted but the number of sessions was adjusted based on unpredicted factors like snow. **(B)** Example of 20 non-rectified waveform averages recorded from a single participant while at rest of soleus MEP following TMS alone **(1)**, soleus TEP following transspinal stimulation alone **(2)**, soleus EMG following TMS delivered before transspinal stimulation **(3)**, and soleus EMG following TMS delivered after transspinal stimulation **(4)**. In protocol 3, summation of MEP and TEP compound action potentials resulting in MEP facilitation is evident. In protocol 4, TEP and MEP do not interact and are easily separated in the surface EMG. EMG, electromyogram; MEP, motor evoked potential; TEP, transspinal evoked potential.

This was a single-blind, within-subject, repeated measures randomized clinical trial. Participants completed at least 20 1-h locomotor training sessions with thoracolumbar transspinal stimulation paired with TMS delivered during assisted stepping in a robotic gait orthosis system (Lokomat 6 Pro®, Hocoma, Switzerland; Figure 1A). Participants were randomized to receive either transspinal-TMS PAS (*N* = 8) or TMS-transspinal PAS (*N* = 6) during locomotor training (Figure 1B; Table 1). Participants were blinded to the PAS protocol. It was difficult for the subject to distinguish the order of stimuli because the interstimulus interval was small. A subset group of three (3) participants completed both PAS and locomotor training protocols after a 6-month washout period (*N* = 3; identified in Table 1). Other participants were unable to complete both training protocols due to personal reasons. Immediately before and 1 day after the last training session, each participant completed two experimental testing sessions to establish neurophysiological changes. We established soleus H-reflex (reported here), transspinal evoked potentials (TEPs), and flexion reflex (not analyzed yet) excitability at rest and during assisted stepping. The experiments were separated by 24 h in an attempt to prevent fatigue in participants. The American Spinal Injury Association Impairment Scale (AIS) score was assessed by a trained clinician to evaluate the clinical SCI level and severity for each participant before the first experimental testing session and training.

**Table 1:** Participant characteristics.

**Subject ID**	**Gender**	**Age (yrs)**	**Height (cm)**	**Weight (kg)**	**Injury level**	**AIS scale**	**Time after injury (yrs)**	**# of training sessions**	**Etiology**	**Medication**
**Transspinal-TMS PAS and locomotor training**										
LR01**^#^**	M	31	185	59	C4	D	15	30	T	None
LR02	F	21	163	52	T7	D	10	30	NT	None
LR03	M	70	180	70	T6	C	12	20	NT	Linaclotide 290 mg 1xD; Alprazolam.25 mg 1xD
LR04**^#^**	M	60	170	91	C5	C	5	20	T	Aspirin 80 mg 1xD; Oxybutynin 10 mg 1xD; Pravastatin 40 mg 1xD; Pericolace 2xD
LR05	F	33	167	82	T12	A	4	19	T	Amitriptyline 25 mg 1xD; Gabapentin 800 mg 3xD; Tramadol 50 mg 2xD
LR06**^#^**	M	38	176	87	T11	D	10	30	T	Gabapentin 100 mg 1xD; Percocet 10 mg (as needed)
LR07	M	57	181	115	C4	C	7	30	T	Baclofen 10 mg 4xD; Bisacodyl 3xD; Gabapentin 300 mg 2xD; Oxybutynin 10 mg3xD; Oxycodone 10 mg 1xD; Senekot 3xD
LR09	M	37	181	84	C5	B	9	20	T	None
**Mean**	**6M, 2F**	**43.3**	**175.3**	**80.0**				**24.8**		
**SD**		**15.8**	**7.3**	**18.5**				**5.1**		
**TMS-transspinal PAS and locomotor training**										
LR11**^#^**	M	31	185	59	C4	D	15	30	T	None
LR12**^#^**	M	38	176	87	T11	D	10	30	T	Gabapentin 100 mg 1xD; Percocet 10 mg (as needed)
LR14	M	27	189	79	T8	A	3	20	T	Oxybutynin
LR15**^#^**	M	61	170	91	C5	C	5	23	T	Aspirin 81 mg 1xD; Oxybotin 15 mg 2xD; Pravastatin 40 mg 1xD; Pericolace 2xD
LR20	F	57	160	64	C4	C	8	26	T	Acetaminophen 500 mg 4xD; Amitriptyline 10 mg 1xD; Baclofen 20 mg 3xD; Cyclobenzaprine 10 1xD; Oxybutynin 10 mg 1xD
LR21	M	71	172	64	C7	C	3	31	T	Gabapentin 700 mg 3xD; Oxybutynin 10 mg 1xD
**Mean**	**5M, 1F**	**47.5**	**175.3**	**74.0**				**26.6**		
**SD**		**16.4**	**9.6**	**12.3**				**4.1**		

*Injury level corresponds to the neurological level of injury. The American Spinal Cord Injury Impairment Scale (AIS) is indicated for each subject based on sensory and motor evaluation per AIS guidelines. The number of transspinal and transcortical paired associative stimulation and assisted step training sessions is indicated for each participant. Medication was taken at similar times per day. xD, Times daily; M, Male; F, Female; C, Cervical; T, Thoracic; T/NT, traumatic/non-traumatic*.

# *Indicates participants who completed both training groups with a 6-month washout between protocols*.

### Paired Transspinal and Transcortical Stimulation

**Figure 2:**
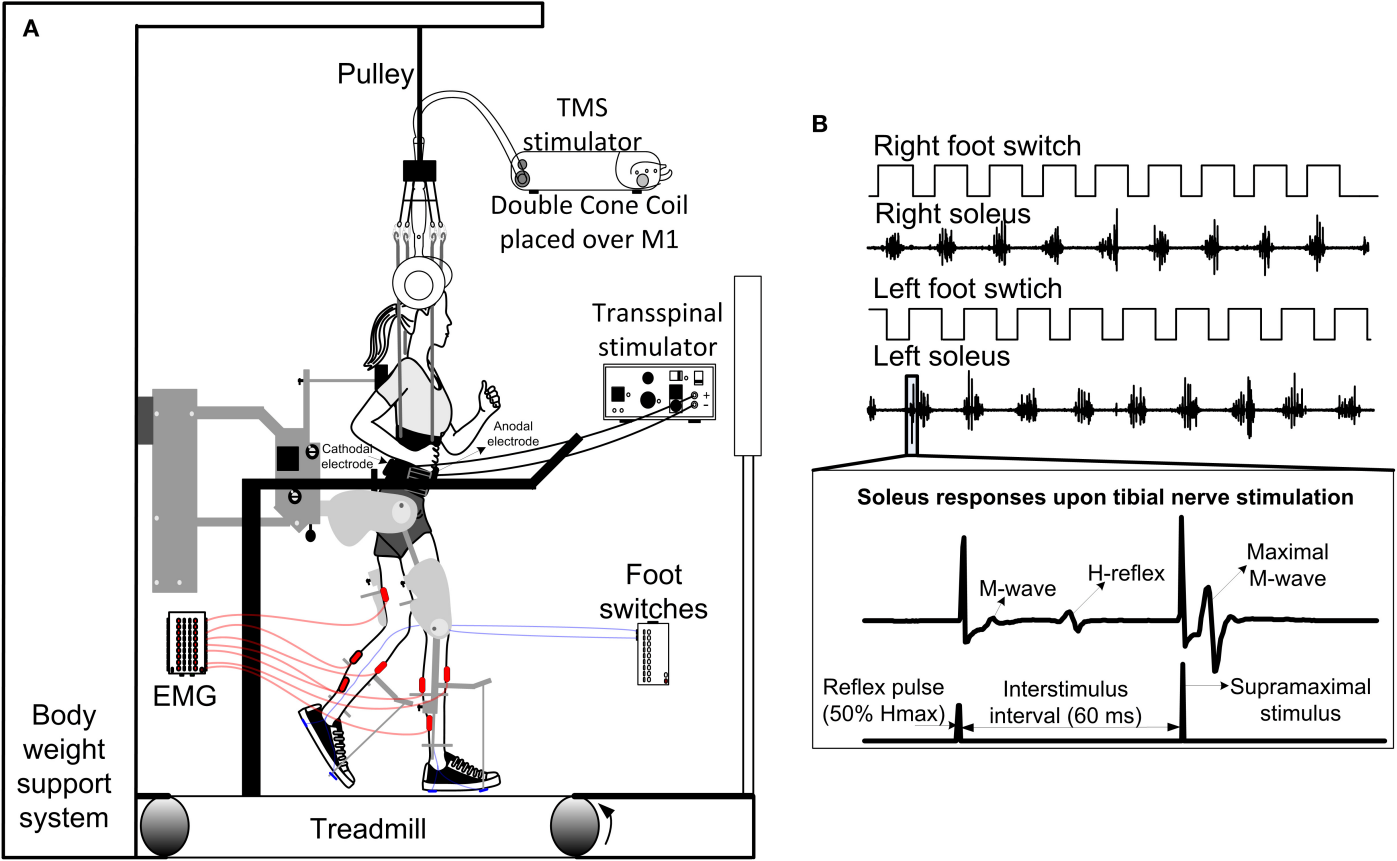
Intervention and experimental protocol during stepping. **(A)** Stimulation of the spinal cord (transspinal) and brain (TMS via a magnetic coil) was delivered during assisted stepping. Subjects stepped with the robotic gait orthosis system while body weight support (BWS) was provided by a harness attached to a pulley. The magnetic coil was held in place using a chin strap and was checked regularly during the training session. Stimuli were triggered based on the signal from the right foot switch registering heel contact and were delivered only during the stance phase. **(B)** Left and right foot switch signals recorded simultaneously with right and left soleus EMG, and stimulation pulses delivered to the posterior tibial nerve. At each bin of the step cycle, a supramaximal stimulus was delivered to the tibial nerve 60 ms after the test stimulus to evoke a maximal M-wave that was used to normalize the associated M-wave and H-reflex at each bin of the step cycle.

During stepping, both transspinal-TMS and TMS-transspinal PAS were delivered during the stance phase of robotic assisted step training (Figure 2A). Paired stimuli were delivered at the mid-stance phase (bins 2–7) based on foot switch signals placed on the leg targeted by TMS (right leg *N* = 9; left leg *N* = 5) (Figure 2B). Paired stimulation was delivered every 2–3 steps, due to the re-charging period of the Magstim stimulator, in blocks of 10-min with by 2-min of assisted stepping without stimulation separating each block. At each training session, a total of 240 paired stimuli were delivered. This resulted in 40-min of paired stimuli delivered during step training, and 20-min of step training without stimulation. Stimuli were delivered at soleus TEP and MEP threshold intensities to minimize perturbations during stepping that may interfere with the robotic assistance.

The interstimulus interval (ISI) was calculated individually for each subject using the relative onset latencies of soleus TEP and soleus MEP (30, 31). The resultant value from Equation (1) was used to deliver transspinal before or after TMS during stepping (31, 32). We adjusted the well-established mathematical estimation of the conduction time to the presynaptic terminals of corticospinal neurons for the first descending motor volley at the spinal cord [MEP-(T_root_+1.5 ms)] (33, 34). The 1.5 ms in Equation (1) allows for synaptic transmission and conduction to the lumbar nerve root at the vertebral foramina (33, 34).

(1) $$\begin{array}{l} \text{ISI}=\text{SOL MEP latency}-\left(\text{SOL TEP latency }+1.5\text{ ms}\right) \end{array}$$

During the transspinal-TMS PAS and locomotor training protocol the ISIs ranged from 14.3 to 17.2 ms (15.6 ± 1.0 ms). Similarly, during the TMS-transspinal PAS and locomotor training protocol ISIs ranged from 13.5 to 18.5 ms (16.2 ± 1.5 ms) across subjects (Table 2). For individuals where MEPs were not evocable/measurable, the ISI was set at 15.5 ms for both protocols based on the mean ISI of measurable responses (*N* = 3). The ISI utilized in the TMS-transspinal PAS protocol allowed TMS-evoked descending motor volleys to arrive at corticospinal presynaptic terminals of spinal motoneurons before transspinal stimulation transynaptically depolarized spinal motoneurons. The ISI in the transspinal-TMS PAS protocol allowed transspinal-induced excitation of dorsal columns to affect TMS-evoked descending motor volleys at their site of origin. The latter is supported by the cortical potentials with an onset latency of 10 ms and duration of 30 ms produced by transspinal stimulation (35).

**Table 2:** Training intervention parameters.

**Subject ID**	**Speed (km/h)**		**BWS (kg)**		**LGF (%)**		**TMS (%MSO)**		**Transspinal (mA)**		**ISI (ms)**
	**Before**	**After**	**Before**	**After**	**Before**	**After**	**Before**	**After**	**Before**	**After**	
**Transspinal-TMS PAS and locomotor training**											
LR01**^#^**	2.2	2.6	21	15	70	50	52	48	238	329	15.9
LR02	1.9	2.1	25	20	90	68	70	58	276	130	16.1
LR03	1.9	2.9	50	51	100	90	58	70	245	300	14.0
LR04**^#^**	1.9	2.6	50	61	100	90	73	78	275	248	17.2
LR05	1.9	2.4	53	52	100	90	62	53	145	170	15.5
LR06**^#^**	2.1	2.2	53	26	90	64	65	62	181	157	16.5
LR07	2.2	2.4	43	43	80	70	46	46	369	344	14.3
LR09	1.5	1.6	68	59	100	95	56	56	236	194	15.5
***t*****-test**	**0.013**		**0.301**		**0.032**		**TMS= 0.394**		**Transspinal= 0.381**		
**TMS-transspinal PAS and locomotor training**											
LR11**^#^**	2.3	2.4	17	6	65	38	70	70	220	568	16.5
LR12**^#^**	2.1	2.1	7	15	70	30	75	76	221	240	16.3
LR14	1.7	1.9	45	40	100	100	NA	NA	180	172	15.5
LR15**^#^**	1.7	1.7	61	43	80	96	88	NA	349	292	16.9
LR20	1.9	1.6	16	23	70	46	55	NA	200	290	13.5
LR21	1.6	1.9	36	33	100	100	55	55	110	180	18.5
***t*****-test**	**0.382**		**0.365**		**0.214**		**TMS= 0.425**		**Transspinal= 0.14**		

*The speed, body weight support (BWS), and leg guidance force (LGF) are indicated for each participant from the first training session (before) to the last training session (after). Transcranial magnetic stimulation (TMS) and transspinal stimulation changed during the intervention is indicated from the first training session (before) to the last training session (after) for each participant. The interstimulus interval (ISI) during the intervention is indicated for each participant. MSO, Maximum Stimulator Output; PAS, paired associative stimulation*.

# *Indicates participants who completed both training groups with a 6-month washout between protocols*.

### Transspinal Stimulation

Transspinal stimulation was delivered based on procedures we have previously used on people with and without SCI (36, 37). A single cathode electrode (Uni-Patch^TM^, 10.2 x 5.1 cm^2^, Wabasha, MA, USA) was placed at the T10 spinous process, identified via palpation and anatomical landmarks, equally between the left and right paravertebrae sides, and secured with Tegaderm transparent film (3M Healthcare, St. Paul, MN, USA). The electrode covered T10 to L1-2 vertebral levels depending on the participant's height. Two interconnected electrodes (anode; same as cathode) were placed on the abdominal muscles or iliac crests, depending on self-reported levels of comfort. Both cathode and anode electrodes were connected to a constant current stimulator (DS7A, Digitimer, Hertfordshire, UK). Stimulation was delivered with a 1-ms monophasic square-wave pulse. The soleus TEP, a spinal evoked compound muscle action potential, threshold was established for each subject during body weight supported (BWS) standing. The soleus TEP threshold was determined as the minimum stimulation intensity required to elicit soleus TEPs of at least 100 μV.

### Transcranial Magnetic Stimulation

TMS was delivered over the primary motor cortex using a Magstim 200 stimulator (Magstim, Whitland, UK) with a double-cone coil (110 mm diameter), orientated to induce a posterior to anterior current flow in the brain. A cap marked with a 7 x 9 cm grid was centered over the vertex, identified by the intersection between the inion and nasion and the left and right ear tragus. The optimal coil position was determined by moving the coil in 1-cm increments. The optimal site corresponded to the largest soleus MEP evoked with submaximal TMS intensity. On average, the center of the double-cone coil was placed 1 cm posterior and 1 cm lateral to the vertex depending on the targeted less impaired leg. The soleus MEP threshold corresponded to the minimum intensity that evoked MEPs of at least 100 μV (38). For participants in which MEPs were not measurable (LR1, LR4, LR6, LR11, LR12, and LR15), the center of the coil was placed directly over the vertex (*N* = 6). To ensure constant coil position during the duration of training, the optimal position, and orientation of the coil was marked on the cap and held in place with a chin strap. This position was checked regularly during training and the optimal stimulation position was re-confirmed at the start of every week along with the MEP threshold.

### Locomotor Training

All participants received BWS assisted step training with the Lokomat for 5 days/week, 1 h/day for 5 weeks (25.8 ± 4.8 sessions; mean ± SD). Over the course of training, the BWS, toe-strap assistance, and leg guidance force (LGF) were adjusted based on the clinical algorithm we previously used for locomotor training in humans with SCI (12, 39). The tension of the toe straps was adjusted based on the left and right tibialis anterior (TA) muscle strength evaluated at the end of each week. BWS and leg guidance force were adjusted based on presence or absence of knee buckling during standing or ankle rolling during stepping. In Table 2, the BWS, LGF and treadmill speed before and after the intervention for each participant is indicated.

### Locomotor EMG Activity Recordings

Following standard skin preparation, surface EMG activity during standing and assisted stepping was recorded from both legs via single bipolar differential electrodes (Motion Lab Systems Inc., Baton Rouge, LA) from the soleus and TA, peroneus longus (PL), medial gastrocnemius (MG), vastus lateralis (VL), vastus medialis (VM), hamstrings (HAM), and gracilis (GRC) muscles. The electrodes were maintained in place by Tegaderm transparent film (3M Healthcare, St Paul, MN, USA). EMG signals were amplified and filtered at frequencies between 10 and 1,000 Hz, sampled at 2,000 Hz using a data acquisition card (National Instruments, Austin, TX, USA), and saved in a personal computer for off-line analysis. Locomotor EMG activity and soleus H-reflexes during assisted stepping were recorded before (baseline) and 1-day after training at similar individualized settings (i.e., BWS, leg guidance force, and treadmill speed) used at baseline.

### Soleus H-Reflexes Recorded During Stepping Before and After Intervention

The soleus H-reflex was evoked according to methods we have previously employed in individuals with and without SCI (8, 12, 40, 41). Soleus H-reflexes were evoked from both left and right legs to assess differences between limbs after SCI (39). With subjects seated and both feet supported by a footrest, a stainless-steel plate of 4 cm^2^ in diameter (anode electrode) was secured approximately 1 cm proximal to the patella. The optimal stimulation site of the posterior tibial nerve (PTN) was probed with a 1-ms monophasic square-wave pulse via a hand-held monopolar stainless steel head electrode (40), and corresponded to the site where the H-reflex could be elicited without a preceding M-wave at low stimulation intensities and when stimulation intensity was increased the M-wave had a similar shape to that of the H-reflex. When the optimal site was identified, the monopolar electrode was replaced by a pre-gelled disposable electrode (SureTrace, Conmed, NY, USA) that was maintained under constant pressure throughout the experiment with athletic foam pre-wrap. The stimulation site was reconfirmed for the permanent monopolar cathode electrode, based on the previously described criteria.

During standing with BWS as needed to avoid knee joint buckling, the soleus H-reflex and M-wave recruitment input-output curves were assembled by sending approximately 80 stimuli at a range of intensities to the PTN at 0.2 Hz. Then, each participant stepped with the assistance of the Lokomat 6 Pro®, and soleus H-reflexes were recorded randomly across the step cycle which was divided into 16 equal time bins (8, 12, 40). During stepping, a supramaximal PTN stimulation was delivered 60 ms after the test stimuli at each bin allowing intensity adjustments in real-time to evoke H-reflexes with corresponding M-waves between 0 and 10% of the maximal M-wave (Mmax) values (Figure 2B) and to ensure constant stimulation parameters during stepping.

These adjustments were made using a custom LabView self-teaching algorithm with respect to stimulation intensities evoking H-reflexes on the ascending limb of the recruitment curve obtained from standing. PTN stimulation was triggered based on signals from the left or right foot switch (MA153, Motion Lab Systems Inc., Baton Rouge, LA, USA). Stimulation was delivered randomly across different phases of the entire step cycle for each subject. The step cycle was divided into 16 equal bins where bin 1 corresponded to heel contact, bin 8 to stance-to-swing transition, bin 9 to swing phase initiation, and bin 16 to swing-to-stance transition. Soleus H-reflex was evoked randomly at different bins once every 2–3 steps. The soleus H-reflex in the transspinal-TMS PAS protocol was recorded in 6 subjects from the left leg and 6 subjects from the right leg. The soleus H-reflex in the TMS-transspinal PAS protocol was recorded in 3 subjects from the left leg and 5 subjects from the right leg. We recorded H-reflexes from the left and right leg because soleus H-reflex reorganization after locomotor training is leg-side dependent in people with SCI, likely to different reorganization of commissural interneurons (2, 4, 12).

### Data Analysis

The soleus H-reflex, M-wave, and maximal M-wave (Mmax) were measured as peak-to-peak amplitude. For each subject, M-waves and H-reflexes recorded during stepping were normalized to the Mmax evoked 60 ms after the test stimuli. H-reflexes were accepted when the preceding M-wave ranged from 0 to 10% of the Mmax. Accepted H-reflexes were averaged at each bin of the step cycle. The mean amplitude of the M-waves and H-reflexes recorded before and after training from each subject were grouped based on the bin number of the step. Statistically significant differences were established with repeated measures analysis of variance (ANOVA) at 16 (bins) times 2 (time of testing) levels. This was done separately for H-reflexes recorded from the left and right legs, and for H-reflexes grouped based on the TMS targeted leg of within the paired protocol.

For each subject, the soleus H-reflex and M-wave recorded at varying stimulation intensities (input-output curve) during BWS standing were normalized to the associated Mmax to counteract for differences of muscle geometry across subjects (40, 42). Then, a sigmoid function was fit to the full soleus M-wave input-output (43, 44). The stimulation intensity value corresponding to 50% of Mmax (S50-Mmax), derived from the sigmoid function, was then used to normalize the stimulation intensities that the H-reflexes were evoked. Averages of normalized H-reflexes and M-waves were calculated in steps of 0.05 (up to 2.0 times the 50% Mmax threshold) and 0.1 (>2.0 times the 50% Mmax threshold). The off-line analysis described previously was done separately for each H-reflex input-output curve of each subject assembled during standing before and after training (4, 8, 41, 45). This analysis was conducted separately for the left and right leg for each protocol, and for H-reflexes grouped based on the TMS targeted leg within the paired protocol.

The background soleus EMG activity for each bin was estimated from the mean value of the rectified and filtered EMG for a duration of 50 ms (high-pass filtered at 20 Hz, rectified, and low-pass filtered at 400 Hz), beginning 100 ms before PTN stimulation. The mean amplitude of the soleus H-reflex was plotted on the *y*-axis vs. the soleus background activity (normalized to the maximal control EMG) on the *x*-axis, and a linear least-square regression was fitted to the data. This analysis was conducted separately for each subject and for the pool data.

The EMG signals from the left and right leg muscles were digitally band-pass filtered at 40–500 Hz and full wave rectified. Then, linear EMG envelopes were obtained at 10 Hz low-pass filter and averaged over 50 steps using the related foot switch signals from the left and right feet. This processing was indifferent between the stepping EMG signals before and after training. The linear EMG envelope of each muscle and individual, was normalized to their maximal homologous EMG during stepping obtained before training. In all statistical tests, significant differences were established at 95% of confidence level. Results are presented as mean values along with the standard error of the mean (SEM).

## Results

### Participants

Fourteen individuals (3 females, 11 males) between the ages of 21 and 71 years of age with chronic (>12 months) SCI participated in this study (Table 1). The level of injury ranged from C4 to T11 and based on the AIS scale, 5 individuals had a neurological deficit classified as AIS D, 6 were AIS C, 1 was AIS B, and 2 were AIS A (Table 1).

### Reorganization of Soleus H-Reflexes Amplitude Modulation After Transspinal/TMS PAS and Locomotor Training During Assisted Stepping

**Figure 3:**
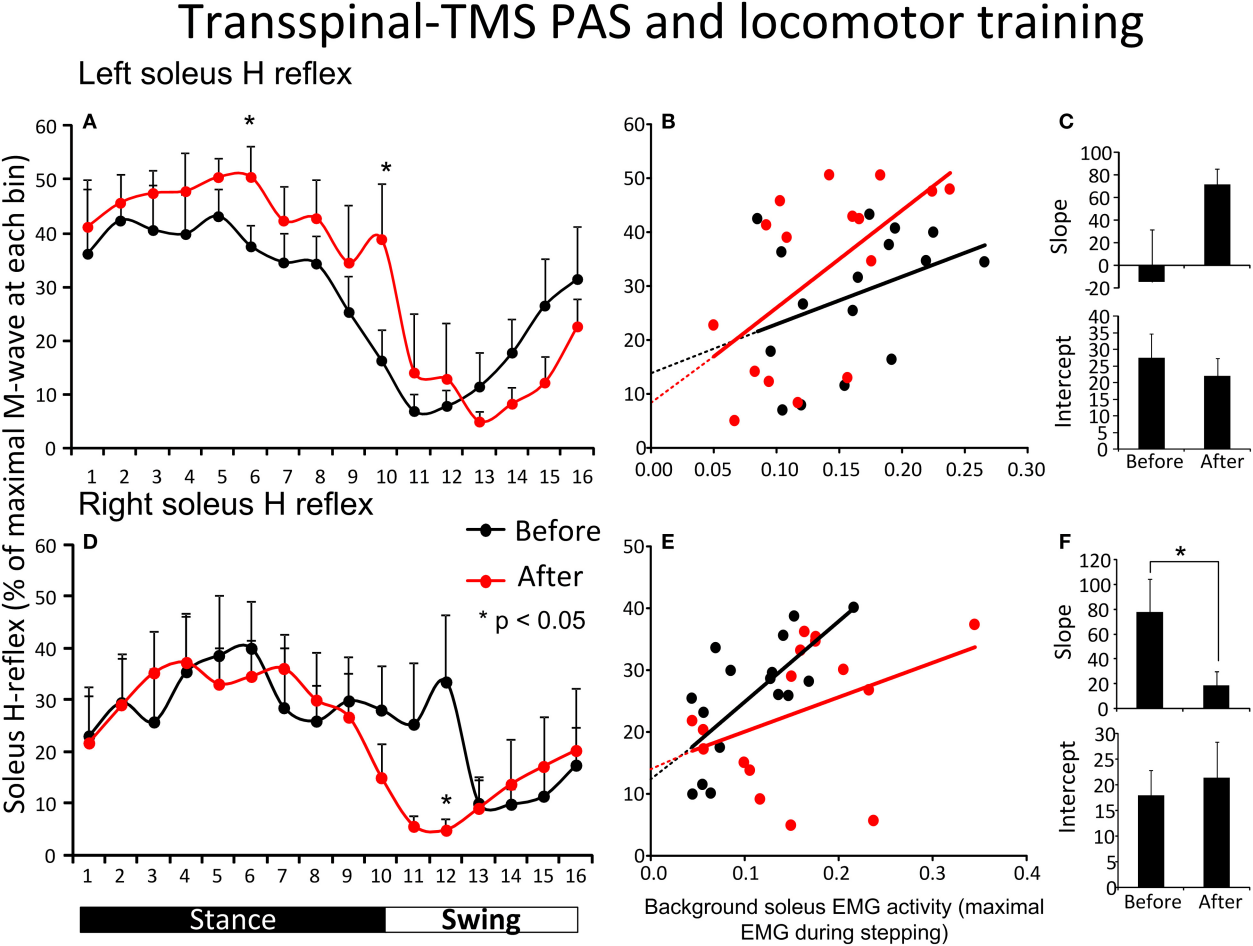
Soleus H-reflex modulation during assisted stepping before and after transspinal-TMS PAS and locomotor training. The mean soleus H-reflex amplitude recorded from the left and right legs **(A,D)** before (black lines) and after (red lines) transspinal-TMS PAS and locomotor training, along with the soleus H-reflex amplitude plotted against the soleus EMG background activity **(B,E)**. The 16 points in graphs **(B,E)** correspond to the 16 bins of the step cycle. The overall amplitude of the slope and intercept for the left **(C)** and right **(F)** legs resulting from the linear relationship between the mean amplitude of the soleus H-reflex and EMG background. EMG, electromyogram; PAS, paired associative stimulation; TMS, transcranial magnetic stimulation. Error bars indicate the SEM. *p < 0.05.

Figure 3A shows the average amplitude of the soleus H-reflex recorded from the left leg, irrespective of the leg targeted by TMS, during assisted stepping at each bin of the step cycle before and after transspinal-TMS PAS and locomotor training. The soleus H-reflex amplitude (Figure 3A) was significantly different across the bins of the step cycle [*F*_(15,155)_ = 7.72, *p* < 0.001] but not over time [*F*_(1,155)_ = 2.52, *p* = 0.114]. No significant interaction was found between the soleus H-reflex amplitude at different bins of the step cycle and time of testing [*F*_(15)_ = 0.85, *p* = 0.61]. We should note, however, that Holm-Sidak pairwise multiple comparisons showed a significant difference in soleus H-reflex amplitudes before and after treatment at bin 6 (*t* = 4.94, *p* < 0.001) and bin 10 (*t* = 2.26, *p* = 0.02). The soleus H-reflex amplitude during stepping was moderately linearly related to the SOL background EMG activity before (*R*^2^ = 0.13) and after (*R*^2^ = 0.35) transspinal-TMS PAS and locomotor training (Figure 3B). The slope and intercept of the linear relationship between the soleus H-reflex amplitude and soleus background EMG activity were not significantly different before and after transspinal-TMS PAS and locomotor training (Figure 3C), supporting for absent changes in reflex gain or reflex threshold.

Figure 3D shows the average amplitude of the soleus H-reflex recorded from the right leg, irrespective of the leg targeted by TMS, during assisted stepping at each bin of the step cycle before and after transspinal-TMS PAS and locomotor training. The soleus H-reflex amplitude was modulated based on the bins of the step cycle [*F*_(15,149)_ = 2.34, *p* = 0.005] but not as a function of time [*F*_(1,149)_ = 0.8, *p* = 0.37]. No significant interactions were found between the soleus H-reflex amplitude at different bins of the step cycle and time of testing [*F*_(15)_= 0.7, *p* = 0.77]. However, we should note that Holm-Sidak pairwise multiple comparisons showed a significant difference in soleus H-reflex amplitudes before and after training at bin 12 (*t* = 2.35, *p* = 0.02). The soleus H-reflex amplitude during stepping was moderately linearly related to the soleus background EMG activity before (*R*^2^ = 0.5) and after (*R*^2^ = 0.15) training (Figure 3E). The slope of the linear relationship between the soleus H-reflex amplitude and background EMG activity was significantly decreased after transspinal-TMS PAS and locomotor training (*p* = 0.03; Figure 3F), indicating a change in reflex gain. The intercept corresponding to the reflex threshold remained unaltered (*p* = 0.35; Figure 3F).

**Figure 4:**
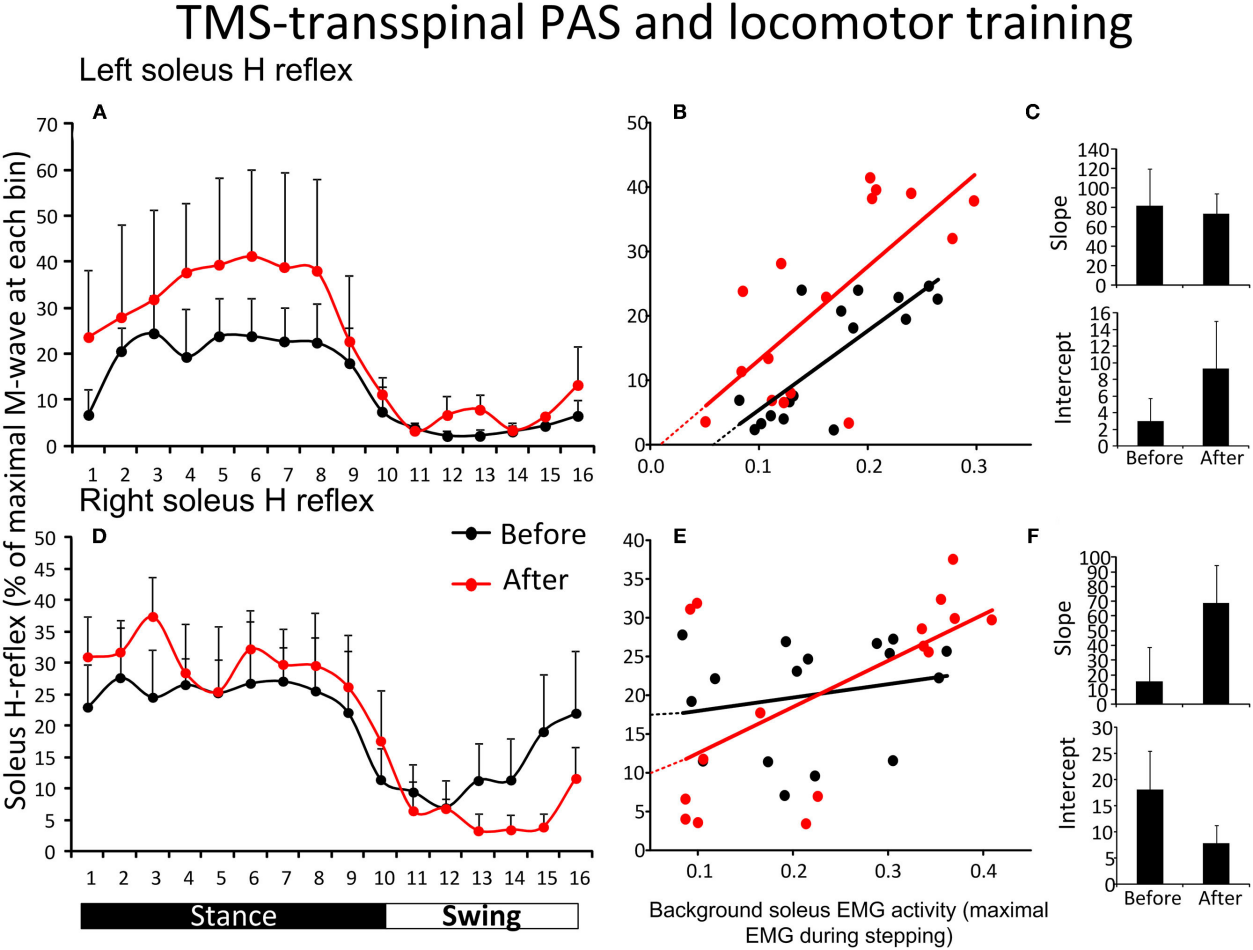
Soleus H-reflex modulation during assisted stepping before and after TMS-transspinal PAS and locomotor training. The mean soleus H-reflex amplitude recorded from the left and right legs **(A,D)** before (black lines) and after (red lines) TMS-transspinal PAS and locomotor training, along with the soleus H-reflex amplitude plotted against the soleus EMG background activity **(B,E)**. The 16 points in graphs **(B,E)** correspond to the 16 bins of the step cycle. The overall amplitude of the slope and intercept for the left **(C)** and right **(F)** legs resulting from the linear relationship between the mean amplitude of the soleus H-reflex and EMG background. EMG, electromyogram; PAS, paired associative stimulation; TMS, transcranial magnetic stimulation. Error bars indicate the SEM.

Figure 4A shows the average amplitude of the SOL H-reflex recorded from the left leg during assisted stepping at each bin of the step cycle before and after TMS-transspinal PAS and locomotor training. The soleus H-reflex amplitude (Figure 4A) was modulated as a function of the step cycle bins [*F*_(15,61)_ = 2.31, *p* = 0.011] and time of testing [*F*_(1,61)_ = 5.16, *p* = 0.027], however, no significant interaction was found [*F*_(15)_ = 0.17, *p* = 1.00]. Holm-Sidak pairwise multiple comparisons showed significant overall differences of means over time (*t* = 2.27, *p* = 0.027). The soleus H-reflex amplitude during stepping showed a moderately positive linear relationship to the soleus background EMG activity before (*R*^2^ = 0.61) and after (*R*^2^ = 0.51) training (Figure 4B). The slope and intercept of the linear relationship between the soleus H-reflex amplitude and background EMG activity were not significantly different before and after TMS-transspinal PAS and locomotor training (Figure 4C), indicating no change in reflex gain or reflex threshold.

Figure 4D shows the average amplitude of the soleus H-reflex recorded from the right leg during assisted stepping at each bin of the step cycle before and after TMS-transspinal PAS and locomotor training. The soleus H-reflex amplitude (Figure 4D) was modulated as a function of the step cycle bins [*F*_(15,125)_ = 3.82, *p* < 0.001] but not as a function of time [*F*_(1,125)_ = 0.01, *p* = 0.89]. No interaction effect was found between the soleus H-reflex amplitude at different bins of the step cycle and time of testing [*F*_(15)_ = 0.59, *p* = 0.87]. The soleus H-reflex amplitude during stepping was minimally linearly related to the soleus background EMG activity before (*R*^2^ = 0.04) and moderately related after (*R*^2^ = 0.36) training (Figure 4E). The slope and intercept of the linear relationship between the H-reflex amplitude and soleus background EMG activity were not significantly different before and after TMS-transspinal PAS and locomotor training (Figure 4F), indicating no changes in reflex gain or reflex threshold.

**Figure 5:**
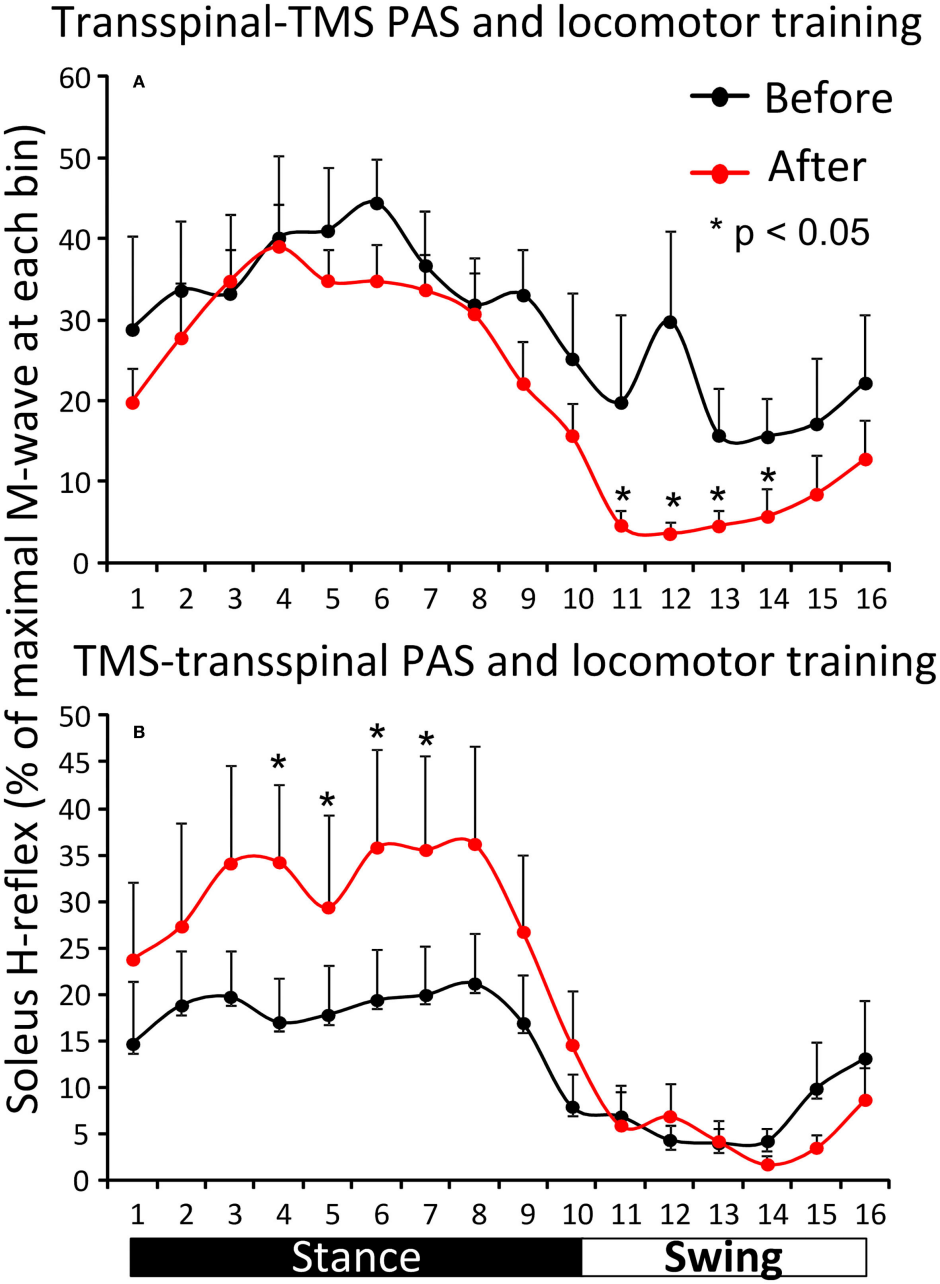
Effects of transspinal and TMS PAS and locomotor training on soleus H-reflex during assisted stepping. Overall amplitude of the soleus H-reflex before and after transspinal-TMS PAS **(A)** and TMS-transspinal PAS **(B)** and locomotor training grouped for each protocol based on the targeted less impaired leg by TMS. The step cycle was divided into 16 equal bins. Bin 1 corresponds to heel strike. Bins 8, 9, and 16 correspond approximately to stance-to-swing transition, swing phase initiation, and swing-to-stance transition, respectively. PAS, paired associative stimulation; TMS, transcranial magnetic stimulation. Error bars indicate the SEM. *p < 0.05.

Figure 5 shows the overall average amplitude of the soleus H-reflex recorded during assisted stepping at each bin of the step cycle grouped based on the targeted leg by TMS at each training session. The soleus H-reflex amplitude before and after transspinal-TMS PAS and locomotor training (Figure 5A) was modulated as a function of the step cycle bins [*F*_(15,180)_ = 5.08, *p* < 0.001] and time of testing [*F*_(1,180)_ = 20.93, *p* < 0.001]. Holm-Sidak pairwise multiple comparisons showed significant different soleus H-reflex amplitudes before and after training (*p* = 0.005) at bins 11, 12, 13, and 14 indicating a return of spinal reflex inhibition during the swing phase after transspinal-TMS PAS and locomotor training (Figure 5A). The soleus H-reflex amplitude before and after TMS-transspinal PAS and locomotor training (Figure 5B) was modulated as a function of the step cycle bins [*F*_(15,154)_ = 5.32, *p* < 0.001] and time of testing [*F*_(1,154)_ = 10.83, *p* < 0.001]. Holm-Sidak pairwise multiple comparisons showed significant different soleus H-reflex amplitudes before and after training (*p* = 0.001) at bins 4, 5, 6, and 7 which implies stability of motoneuronal depolarization during the mid- and late stance phases (Figure 5B).

### Soleus H-Reflex Input-Output Curves After Transspinal/TMS PAS and Locomotor Training

**Figure 6:**
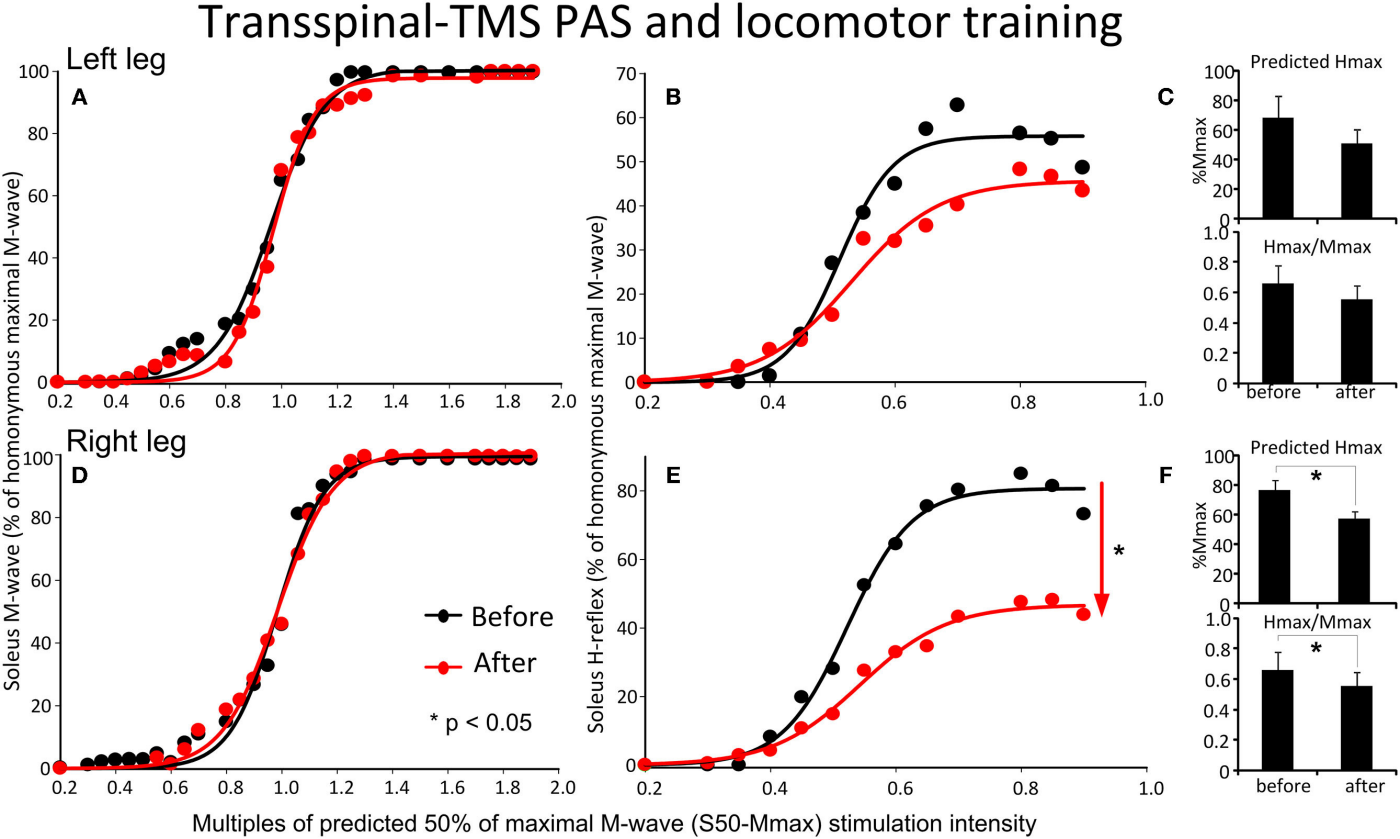
Effects of transspinal-TMS PAS and locomotor training on soleus H-reflex input-output curves. Soleus M-waves and H-reflex recruitment input-output curves recorded at different stimulation intensities from the left **(A,D)** and right **(B,E)** legs during standing with body weight support before and after transspinal-TMS PAS and locomotor training. Sigmoid functions are fitted to the input-output curves. **(C,F)** Corresponding predicted Hmax and Hmax/Mmax for the left **(C)** and right **(F)** legs. Hmax, maximal H-reflex; Mmax, maximal M-wave; PAS, paired associative stimulation; TMS, transcranial magnetic stimulation. Error bars indicate the SEM. *p < 0.05.

The soleus M-waves and H-reflexes recorded at different stimulation intensities from the left and right legs during BWS standing before and after transspinal-TMS PAS and locomotor training and the associated sigmoid fits are shown in Figure 6. A two-way ANOVA showed that normalized soleus M-waves were not statistically significant different before and after training for the left leg [*F*_(1,122)_ = 1.61, *p* = 0.2; Figure 6A) or the right leg [*F*_(1,155)_ = 0.09, *p* = 0.76; Figure 6D]. These results suggest that changes in soleus H-reflex size after training were not due to the recruitment of different motoneurons by the Ia afferent volley, and that stimulation and recording procedures were not different between sessions. In Figures 6B,E, the corresponding normalized soleus H-reflexes are plotted against multiples of the predicted S50-Mmax. The soleus H-reflex was significantly decreased for the right leg [*F*_(1,107)_ = 46.9, *p* < 0.001; Figure 6E], accompanied by a reduced predicted maximal H-reflex (Hmax) and Hmax/Mmax ratio (Figure 6F), but not for the left leg [*F*_(1,94)_ = 3.88, *p* = 0.052; Figures 6B,C] after training. From the estimated sigmoid function parameters only the predicted Hmax was decreased for the right leg while the function m, S50, H-slope, and stimulation thresholds remained unaltered for H-reflexes recorded from either left or right legs during BWS standing (for all *p* > 0.05).

**Figure 7:**
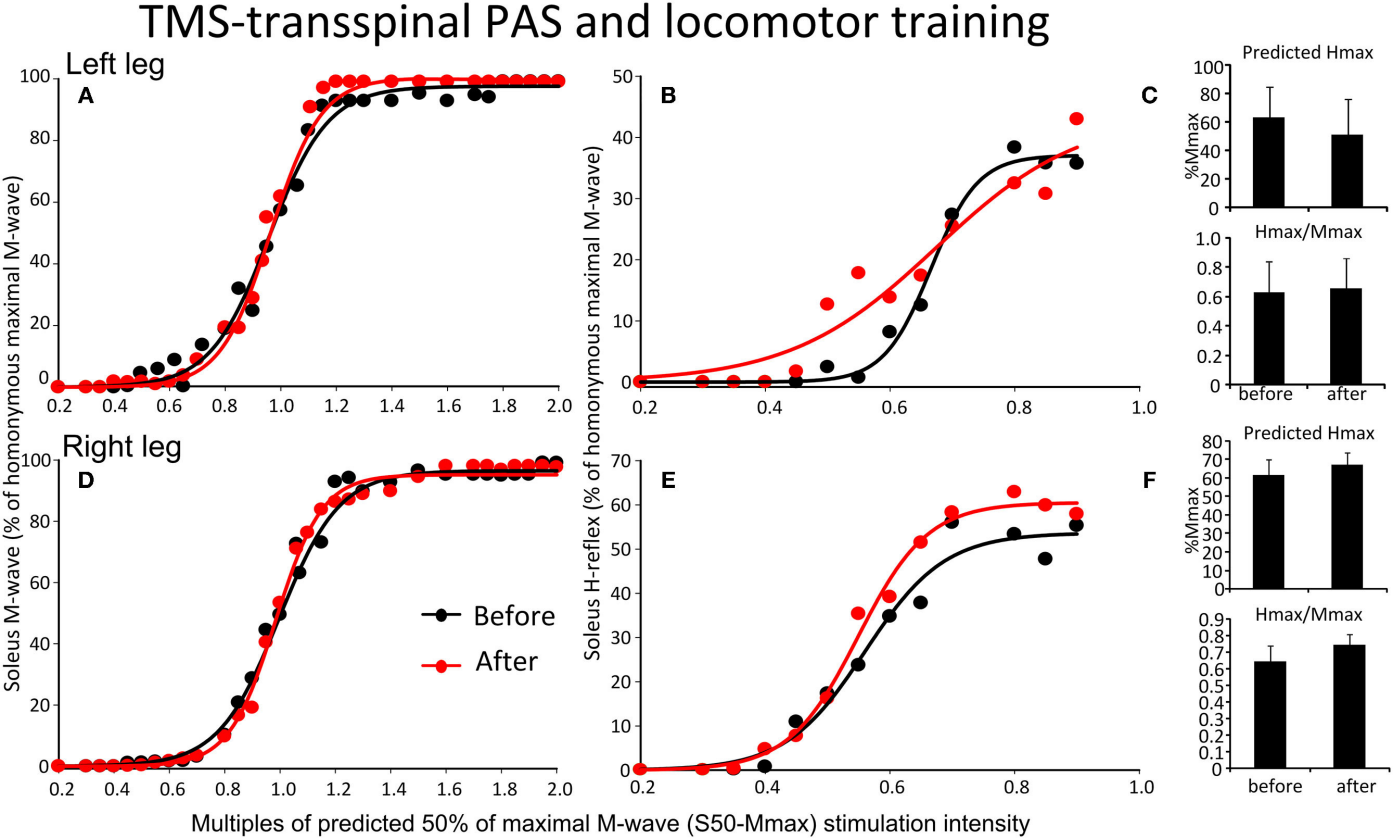
Effects of TMS-transspinal PAS and locomotor training on soleus H-reflex input-output curves. Soleus M-waves and H-reflex recruitment input-output curves recorded at different stimulation intensities from the left **(A,D)** and right **(B,E)** legs during standing with body weight support before and after TMS-transspinal PAS and locomotor training. Sigmoid functions are fitted to the input-output curves. **(C,F)** Corresponding predicted Hmax and Hmax/Mmax for the left **(C)** and right **(F)** legs. Hmax, maximal H-reflex; Mmax, maximal M-wave; PAS, paired associative stimulation; TMS, transcranial magnetic stimulation. Error bars indicate the SEM.

The soleus M-waves and H-reflexes recorded at different stimulation intensities from the left and right legs during BWS standing before and after TMS-transspinal PAS and locomotor training and the associated sigmoid fits are shown in Figure 7. A two-way ANOVA showed that normalized soleus M-waves were not statistically significant different before and after training for the left [*F*_(1,53)_ = 0.41, *p* = 0.52; Figure 7A] or right leg [*F*_(1,134)_ = 0.422, *p* = 0.51; Figure 7D], again, suggesting similar stimulation and recording procedures across different testing sessions. In Figures 7B,E, the corresponding normalized soleus H-reflexes are plotted against multiples of S50-Mmax. No significant differences were found for the H-reflex recorded from the left [*F*_(1,99)_ = 2.87, *p* = 0.093; Figures 7B,C] or right leg [*F*_(1,46)_ = 0.47, *p* = 0.49; Figures 7E,F] after TMS-transspinal PAS and locomotor training. All parameters estimated from the sigmoid function (Hmax, function m, S50, H-slope, and stimulation thresholds) remained unaltered for H-reflexes and M-waves recorded from either left or right legs during BWS standing (for all *p* > 0.05).

**Figure 8:**
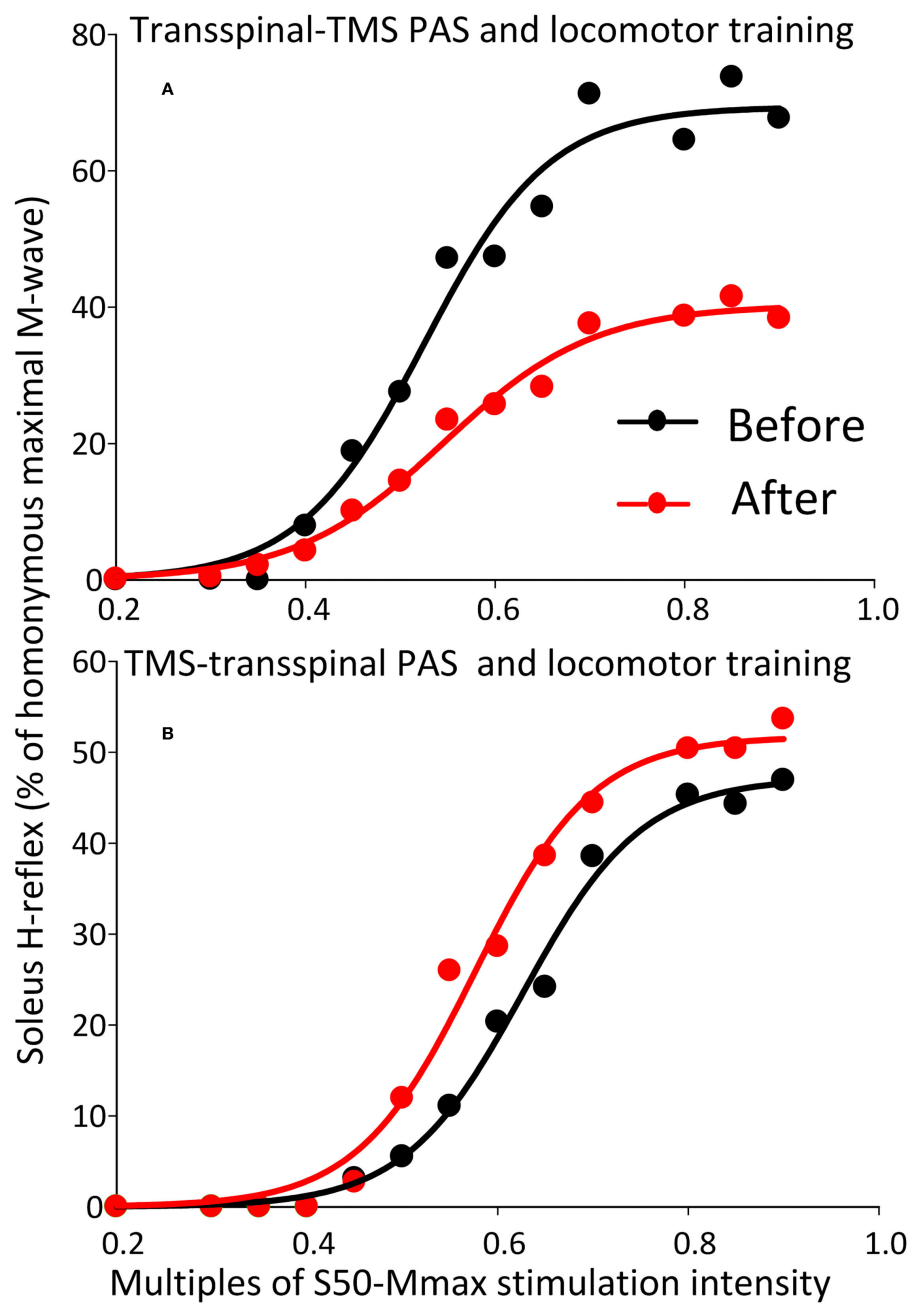
Soleus H-reflex recruitment input-output curves before and after training grouped based on TMS targeted leg. Soleus H-reflex recruitment input-output curves grouped based on the TMS less impaired targeted leg recorded at different stimulation intensities during body weight supported standing before and after transspinal-TMS **(A)** PAS and TMS-transspinal PAS **(B)** and locomotor training. Sigmoid functions are fitted to the input-output curves. PAS, paired associative stimulation; TMS, transcranial magnetic stimulation.

The soleus H-reflex grouped based on TMS targeted leg for M1 stimulation during training was significantly reduced after transspinal-TMS PAS and locomotor training [*F*_(1,119)_ = 28.19, *p* < 0.001; Figure 8A] with significant differences between time found from 0.55 to 0.9 multiples of S50-Mmax. While a tendency for facilitation of H-reflex excitability after TMS-transspinal PAS and locomotor training was present (Figure 8B), the two-way ANOVA showed a non-significant effect between time [*F*_(1,121)_ = 3.1, *p* = 0.08].

### Locomotor EMG Activity After Transspinal/TMS PAS and Locomotor Training

**Figure 9:**
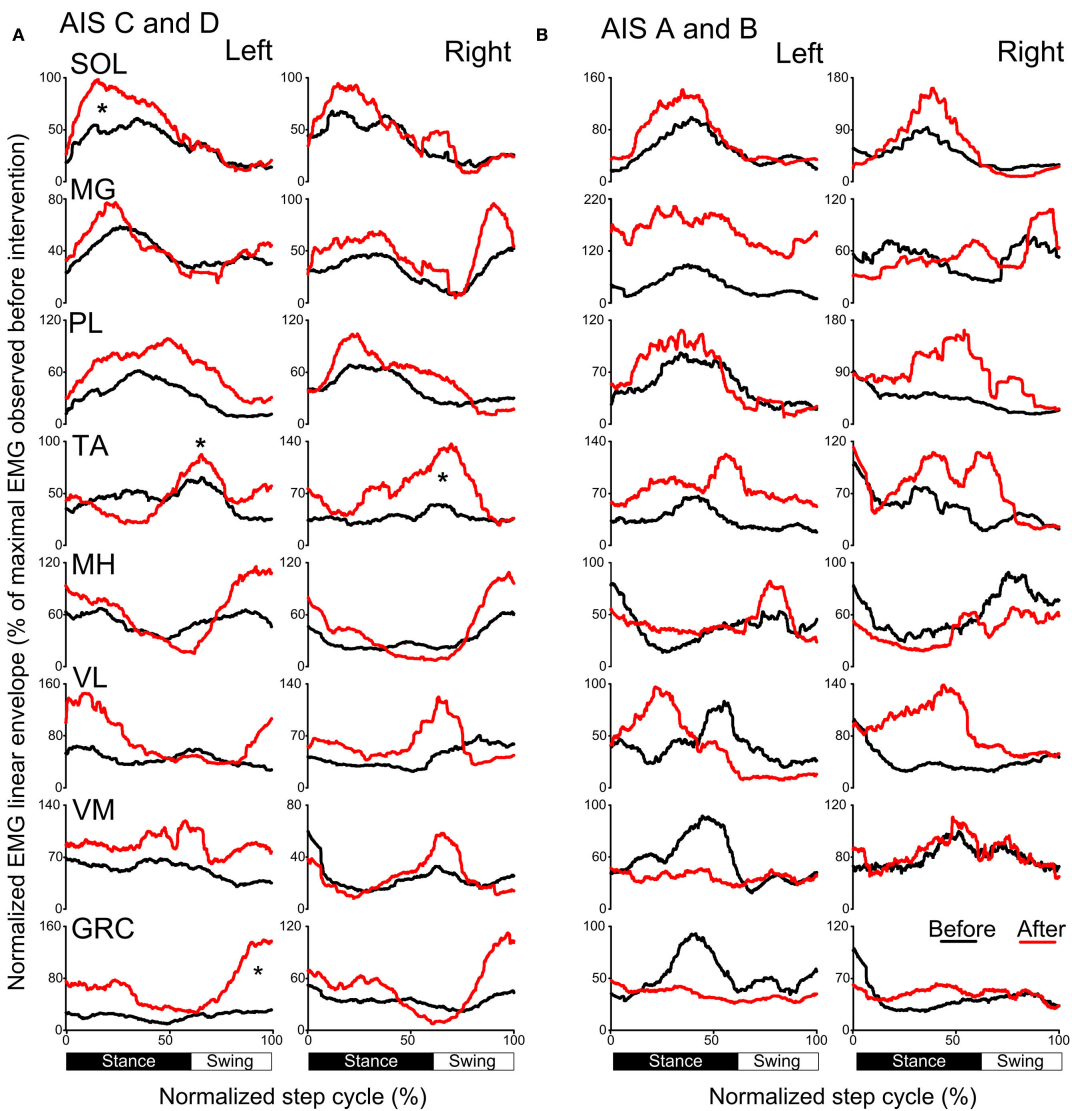
Mean muscle activation pattern before and after transspinal-TMS PAS and locomotor training during assisted stepping. Left and right leg locomotor muscle EMG activity during stepping before (black lines) and after (red lines) transspinal-TMS PAS and locomotor training from participants with incomplete **(A)** and complete SCI **(B)**. EMG is normalized to the maximal homologous EMG obtained during stepping before training. Heel strike is at zero normalized gait cycle. EMG, electromyographic; GRC, gracilis; MH, medial hamstrings; MG, medialis gastrocnemius; PAS, paired associative stimulation; PL, peroneus longus; SOL, soleus; TA, tibialis anterior; TMS, transcranial magnetic stimulation; VL, vastus lateralis; VM, vastus medialis. *p < 0.05.

**Figure 10:**
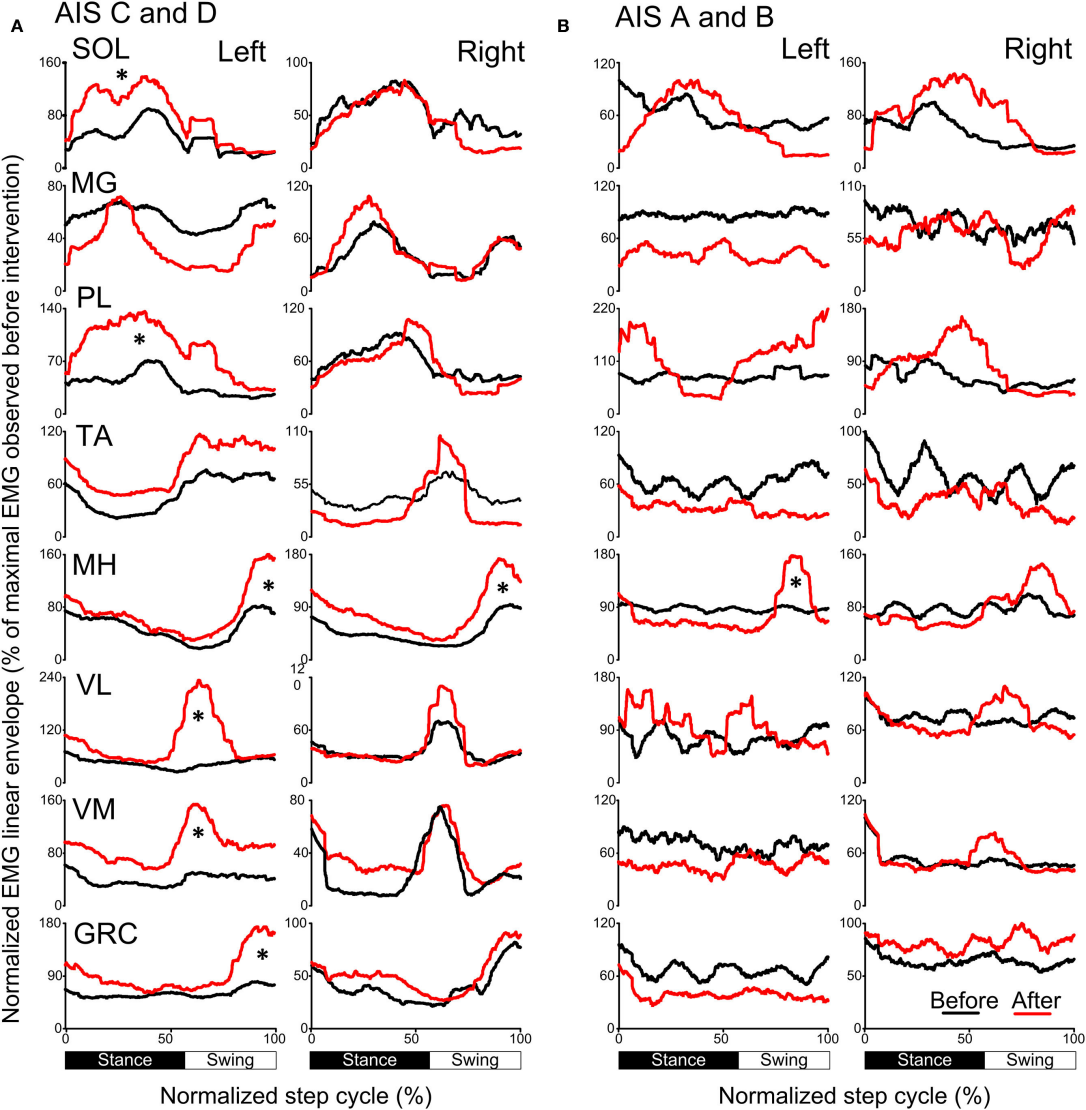
Mean muscle activation pattern before and after TMS-transspinal PAS and locomotor training during assisted stepping. Left and right leg locomotor muscle EMG activity during stepping before (black lines) and after (red lines) TMS-transspinal PAS and locomotor training from participants with incomplete **(A)** and complete SCI **(B)**. EMG is normalized to the maximal homologous EMG obtained during stepping before training. Heel strike is at zero normalized gait cycle. EMG, electromyographic; GRC, gracilis; MH, medial hamstrings; MG, medialis gastrocnemius; PAS, paired associative stimulation; PL, peroneus longus; SOL, soleus; TA, tibialis anterior; TMS, transcranial magnetic stimulation; VL, vastus lateralis; VM, vastus medialis. *p < 0.05.

The activation profiles and amplitude of locomotor EMG activity from left and right legs for subjects with motor incomplete and complete SCI during robotic assisted stepping before and after each intervention paradigm are indicated in Figures 9, 10. Maximal peak-to-peak EMG amplitudes increased following transspinal-TMS PAS and locomotor training for the left SOL (*t* = 2.98, *p* = 0.04), TA (*t* = 5.895, *p* = 0.004), and GRC (*t* = 2.865, *p* = 0.045), and right TA (*t* = 2.877, *p* = 0.034) muscles (Figure 9A). No significant changes were observed in the remaining muscles (*p* > 0.05; Figure 9B). Note that the increased TA EMG activity coincided with significant changes in the amplitude, onset and offset in the ipsilateral knee flexor and knee extensor muscles after training compared with that observed before training (Figure 9). Similar results were observed after TMS-transspinal PAS and locomotor training, during which maximal peak-to-peak EMG amplitudes were increased in incomplete SCI for left SOL and PL and knee flexors and extensors (Figure 10A). An improvement of bilateral SOL and MH EMG amplitude was also observed after the intervention in people with motor complete SCI (Figure 10B). Lastly, it should be mentioned that although changes in the maximal peak-to-peak EMG was not observed, the altered phase-dependent modulation is suggestive of greater motoneuron recruitment and that motoneuron recruitment was relevant to the phase of the step cycle, which is a prerequisite for decreased spastic gait pattern. The changes in motoneuron pool activity after training can be summarized by an increase in EMG amplitude, and restoration of biphasic (when a muscle contracts in more than one phase within a single step cycle) EMG activity.

## Discussion

In this randomized clinical trial, we demonstrated for the first time reported in the literature significant changes in soleus H-reflex phase-dependent amplitude modulation after locomotor training coupled with paired transspinal and TMS in people with chronic motor incomplete and complete SCI. After transspinal-TMS PAS and locomotor training the left soleus H-reflex was facilitated during the mid-stance and early-swing, while the soleus H-reflex in the right leg was depressed during the mid-swing. Further, soleus H-reflex input-output curve was decreased in the right leg at rest. TMS-transspinal PAS and locomotor training did not produce significant changes on the soleus H-reflex during stepping or at rest. When H-reflexes were grouped based on the TMS-targeted leg, transspinal-TMS PAS and locomotor training improved reflex inhibition during the swing phase, while TMS-transspinal PAS and locomotor training improved excitation and stabilization of reflexively induced motoneuronal depolarization during the stance phase. Furthermore, both transspinal-TMS and TMS-transspinal PAS and locomotor training increased EMG amplitude and promoted a more physiological modulation of motor activity and thus depolarization of motoneurons in a pattern that promotes stepping.

During walking, appropriate engagement of spinal neuronal circuits at each phase of the step is reflected by the phase-dependent amplitude modulation of the soleus H-reflex. In neurologically intact individuals, soleus H-reflex amplitudes are minimal during swing and at heel contact, increase rapidly during stance, and decrease abruptly after toe-off (42, 46). SCI disrupts this phase-dependent soleus H-reflex amplitude modulation during walking, varying from relatively normal in some patients to completely absent in others (7, 8, 12, 39, 47). The most common change observed after locomotor training is the partial return of soleus H-reflex depression during the swing phase; but extensor motoneuron excitability during the stance phase remains unstable (12, 39). Consequently, the amplitude modulation of H-reflexes during walking depicts pathological changes of spinal circuits in individuals with SCI, and beneficial circuit reorganization after locomotion-based therapeutic interventions.

For the first time reported in the literature, this clinical trial focused on coupling locomotor training with paired stimulation of the nervous system taking advantage of Hebbian mechanisms of neuroplasticity (33, 48, 49). PAS was timed to occur during the stance phase because transspinal stimulation produces bilateral leg extension at rest and during standing in people with and without SCI (37, 50, 51). In the transspinal-TMS PAS and locomotor training protocol, the transspinal stimulation induced ascending afferent volleys reached primary motor cortex at time that allowed to affect the descending motor volleys at their site of origin (26). This pathway of action is supported by increased corticospinal and decreased spinal reflex excitability and spinal motor output when transspinal-TMS PAS is delivered at rest for 40-min in healthy subjects (30, 31). The increased H-reflex excitability during stance phase in the left leg is suggestive of motoneuronal depolarization potentiation, while the return of spinal inhibition at mid-swing in the right leg (bin 12) suggests for reorganization of reciprocal inhibition pathway between ankle flexors and extensors (Figure 3). Consequently, transspinal stimulation in the transspinal-TMS PAS and locomotor training protocol could have affected descending control of spinal motoneurons and interneurons, especially Ia inhibitory interneurons (52–54), as well as the interneurons engaged in pre-motoneuronal control and phasic presynaptic GABAergic inhibitory action on afferent volleys (2, 55–60). This is clearly evident by the lack of effects following TMS-transspinal PAS and locomotor training protocol (Figure 4), and the differential H-reflex neuroplastic changes when data were grouped based on the targeted TMS leg during the training intervention (Figure 5). The latter suggests that spinal reflex reorganization can result partly from strengthening corticospinal neural connections through PAS protocols.

Stimulation of the brain and spinal cord synchronized to the phase of the step cycle may have the potential to augment the benefits of activity-based therapies and further decrease hyperreflexia and muscle spasticity by restoring pre-motoneuronal inhibitory control, motoneuron excitability through recovery of homeostasis and adjusting neuronal properties, such as threshold excitability state (61). Such mechanisms are also involved in neurophysiological biofeedback training protocols, such as operant conditioning of reflexes that improve locomotion and interlimb coordination (47, 62). Thus, repeated motor activity accomplished by exercise-based or stimulation-based protocols, use similar neuronal pathways enhancing changes in neuronal excitability states as well as the synaptic integration of sensory feedback.

Based on the specific SCI-induced pathological behavior of reflex modulation during stepping transspinal-TMS or TMS-transspinal PAS may provide a targeted intervention. For example, reduced inhibition of the soleus H-reflex during the swing phase, which may cause toe-drag, can be targeted to be reduced by transspinal-TMS PAS. Further, the resemblance between the reflex reorganization observed in this study and locomotor training alone in individuals with SCI (12, 13, 39, 41), support the use of combined interventions that target stimulation of the brain and spinal cord with exercise-based therapies.

Several methodological limitations in the current study warrant consideration. Neurophysiological tests were not performed at different time points following cessation of the intervention. Thus, we cannot comment on the sustainability of neuroplasticity and neurorecovery beyond 1–2 days. Further, participants received an average of 25 sessions. However, a consensus regarding the adequate “dosages” of locomotor training does not exist (63, 64). Future studies are warranted to assess the time course of neuroplasticity while administering more training sessions. In addition, there were no control experiments performed because of the complexity of the research. Possible control experiments for this protocol include brain or transspinal stimulation alone, or locomotor training without stimulation. Our group has collected data from TMS evoked MEPs, but analysis has yet to be completed. Last, both intervention protocols guarantee testing in cases of subacute SCI (65).

It is important to note that the PAS protocol used in the current clinical trial is uniquely different from PAS protocols targeting the brain and peripheral nerves (33, 66, 67). In the current protocol, transspinal instead of peripheral nerve stimulation excited sensory fibers, motoneurons, and spinal interneurons over multiple segments causing widespread activity within the spinal cord affecting intra- and interlimb coordination. In contrast, paired TMS and peripheral nerve protocols target motoneurons and muscles specific to the stimulated peripheral nerve. This specificity of paired TMS and peripheral nerve stimulation may limit the effects to intralimb activity. Implementing PAS protocols during locomotor training, such as in the current study, in rehabilitation clinics may be beneficial to individuals with SCI they are, however, exceedingly difficult from a practical standpoint. Future studies may examine whether priming the nervous system with transspinal-transcortical PAS before locomotor training is equally beneficial.

This study provides evidence that TMS and transspinal PAS during step training alters soleus H-reflex excitability in individuals with chronic SCI. The neurophysiological changes were observed in both limbs even in cases of motor complete SCI. We theorize that TMS and transspinal PAS during stepping can drive neuroplasticity, exclusively by sensory feedback mechanisms, and may strengthen corticospinal connections in neurological disorders characterized by weak or absent corticospinal drive, but more research is needed. Restoration of phase-dependent soleus H-reflex amplitude modulation and EMG amplitude during assisted stepping could potentially facilitate appropriate control of leg muscles during locomotion in this patient population. In conclusion, non-invasive transspinal and TMS PAS warrant further investigation as an intervention that potentially may lead to improvements in leg function and supplement the benefits of locomotor training in people with SCI.

## Data Availability Statement

The raw data supporting the conclusions of this article will be made available by the authors, without undue reservation.

## Ethics Statement

The studies involving human participants were reviewed and approved by Institutional Review Board of the City University of New York. The patients/participants provided their written informed consent to participate in this study.

## Author Contributions

MK: conception and design of research, interpreted results of experiments and wrote first draft of manuscript. TP, MZ, MG, EG, MI, JL, LM, and MK: performed experiments. MZ, MG, EG, MI, and MK: analyzed data. MI and MK: prepared figures. TP, LM, and MK: edited and revised manuscript. TP, MZ, MG, EG, MI, JL, LM, and MK: approved final version of manuscript. All authors contributed to the article and approved the submitted version.

## Conflict of Interest

The authors declare that the research was conducted in the absence of any commercial or financial relationships that could be construed as a potential conflict of interest.
